# Whole-genome sequence analysis for evaluating the safety and probiotic potential of *Lactiplantibacillus pentosus* 9D3, a gamma-aminobutyric acid (GABA)-producing strain isolated from Thai pickled weed

**DOI:** 10.3389/fmicb.2022.969548

**Published:** 2022-09-23

**Authors:** Nachon Raethong, Chalat Santivarangkna, Wonnop Visessanguan, Pannita Santiyanont, Wuttichai Mhuantong, Nipa Chokesajjawatee

**Affiliations:** ^1^Institute of Nutrition, Mahidol University, Nakhon Pathom, Thailand; ^2^National Center for Genetic Engineering and Biotechnology (BIOTEC), Pathum Thani, Thailand

**Keywords:** whole-genome sequence, *Lactiplantibacillus pentosus*, GABA-producing strain, safety evaluation, probiotic

## Abstract

*Lactiplantibacillus pentosus* 9D3, a prominent gamma-aminobutyric acid (GABA)-producing bacteria isolated from Thai pickled weed was characterized for its safety and probiotic properties *via* whole-genome analysis and *in vitro* testing. The whole-genome sequence of *L. pentosus* 9D3 was determined using a hybrid-sequencing approach, combining PacBio and Illumina technologies. A 3.81-Mbp genome of *L. pentosus* 9D3 consisting of one 3.65-Mbp chromosome and six plasmids (1.9–71.9 Kbp) was identified with an estimated GC content of 46.09% and 3,456 predicted genes. The strain was confirmed to be *Lactiplantibacillus pentosus* according to the high average nucleotide identity value of >95% and digital DNA–DNA hybridization scores of >70% to the *L. pentosus* type strain. Comparative genome analysis with other *L. pentosus* strains showed that the GABA-producing capability was specific to the strain 9D3. Genes related to GABA biosynthesis and transport were identified on a plasmid, pLPE-70K, indicating the acquired nature of this property. The safety of *L. pentosus* 9D3 was demonstrated through the lack of genes related to the production of toxins, biogenic amines, and antimicrobial drugs. Although the strain exhibited resistance to ampicillin and chloramphenicol, none of the antimicrobial resistance (AMR) genes were associated with mobile elements, i.e., plasmids and prophages. Therefore, the strain is considered to have low risk of transferring the AMR genes to other, potentially pathogenic bacteria. In addition, *L. pentosus* 9D3 showed good survivability in the gastrointestinal tract environment and was able to adhere to the intestinal cell *in vitro.* Therefore, *L. pentosus* 9D3 is concluded to be safe, with the potential to be used as a probiotic, exerting its health benefit through GABA production in the food system. The GABA-producing capability of the strain *in vivo* is the subject of further investigation.

## Introduction

Lactic acid bacteria (LAB) are biotechnologically important groups of gram-positive bacteria that are non-motile and tolerant to low pH. They usually produce lactic acid as the common metabolic end product of carbohydrate fermentation (Wang et al., 2021). Many of them are considered probiotics due to their potential health benefits related to the gut microbiome, gut function, and immune system (Hajela et al., 2015). *Lactiplantibacillus pentosus* (previously known as *Lactobacillus pentosus*) has gained increasing attention due to its immunostimulatory activity, cholesterol-lowering property, and gamma-aminobutyric acid (GABA)-producing capacity (Izumo et al., 2010; Kotani et al., 2010; Bendali et al., 2017; Kittibunchakul et al., 2021; Phuengjayaem et al., 2021). GABA is an inhibitory neurotransmitter that has been reported to be associated with anti-obesity and reverse diabetes (Soltani et al., 2011; Sato et al., 2021). A previous study by Patterson et al. (2019) showed that dietary administration of GABA-producing LAB could improve metabolism and depressive-like behavior in a mouse model (Patterson et al., 2019). Our previous study screened LAB from Thai fermented foods for their ability to grow in brown rice milk and produce GABA (Kittibunchakul et al., 2021). The results showed that *L. pentosus* 9D3, a GABA-producing strain isolated from pickled weeds, was able to produce high levels of GABA. The 9D3 strain was successfully used to create a plant-based GABA drink using brown rice as a raw material. Additionally, the rice milk fermented with *L. pentosus* 9D3 contained a high number of live bacteria (8.6 log CFU/ml; Kittibunchakul et al., 2021) prompting the drink to be classified as a probiotic drink, if the strain was characterized as probiotic. Thus, the safety and probiotic properties of the strain 9D3 should be thoroughly investigated.

Previous studies used the advent of whole-genome sequencing technologies and bioinformatics for evaluating the safety and potential benefits of the microorganisms used in fermented food such as fermented pork and fermented olives (Abriouel et al., 2017a; Chokesajjawatee et al., 2020). Therefore, this study aimed to sequence the *L. pentosus* 9D3 genome and evaluate the safety profile and probiotic properties of the strain. For safety evaluation, we followed the procedural guideline proposed by Chokesajjawatee et al. (2020). The guideline provides details on microbial strain safety assessment *via* whole-genome sequence (WGS) analysis, including the selection of appropriate sequencing platforms, unambiguous species identification, identification of virulence and undesirable genes, identification of functional and transferrable antimicrobial resistance (AMR) genes, and identification of antimicrobial drug production capability. This guideline was based on a decision tree for microbial safety evaluation proposed by Pariza et al. (2015), in which the rationale for each assessment step was provided in detail. For probiotic properties, the strain’s ability to survive conditions mimicking *in vitro* digestion and adhesion to the intestinal cell line was analyzed to determine the strain’s probiotic potential (Hill et al., 2014).

## Materials and methods

### Culture condition for DNA extraction

*Lactiplantibacillus pentosus* 9D3 was obtained from the culture collection of the Institute of Nutrition, Mahidol University, Thailand. The bacterium was stored at – 80°C in de Man, Rogosa and Sharp (MRS) broth containing 20% glycerol. To obtain cells for DNA extraction, the strain was grown on MRS agar (Difco Laboratories Inc., United States) and incubated at 30°C for 1–2 days. Then, the purified single colony was sub-cultured into 5 ml MRS broth and incubated at 30°C for 12–16 h. The cell pellet obtained from the fresh overnight culture was used for DNA extraction.

### Genomic and plasmid DNA extraction

Genomic DNA was extracted using a Wizard Genomic DNA Purification Kit (Promega Corporation, United States) according to the manufacturer’s instructions with some modifications as recommended by Chokesajjawatee et al. (2020). The DNA pellet was dissolved in DNase/RNase-free water. The concentration and purity of the DNA were measured using a NanoDrop ND-1000 spectrophotometer (Thermo Fisher Scientific, United States). The integrity of the gDNA was assessed using agarose gel electrophoresis. Intact, high purity gDNA with an OD_260_/OD_280_ ratio of 1.8–2.0 and an OD_260_/OD_230_ ratio of 2.0–2.2 was used for whole-genome sequencing. The plasmid DNA was extracted using a ZymoPURE Plasmid Miniprep kit (Zymo Research Corporation, CA, United States) with some modifications as recommended by Chokesajjawatee et al., 2020. The existence and size of the plasmid, if present, were determined using 0.5% agarose gel electrophoresis.

### WGS and *de novo* assembly process

The gDNA was subjected to two complementary next-generation sequencing platforms, i.e., long-read and short-read sequencing. For long-read sequencing, the gDNA was sequenced using PacBio RSII cell by a certified service provider (DNA Link, Inc., Republic of Korea). For short-read sequencing, the gDNA was sequenced using Illumina HiSeq2000 by a service provided by Macrogen Inc., (Republic of Korea). Several assembly protocols were tested, and the protocol that provided the most accurate (based on manual investigation of the actual reads in problematic areas) and complete (the ability to detect the smallest plasmid presence in the genome) genome was selected. Firstly, the genome was assembled from the long-read sequencing data using the Unicycler pipeline involving the Miniasm assembler and Racon 2 polishing process (Wick et al., 2017). Next, the base accuracy was improved by the short-read sequencing data using the Snippy base-correction protocol (Seemann, 2015). The quality of genome assembly was assessed in terms of the continuity and completeness of orthologous genes using QUAST version 5.0 (Gurevich et al., 2013) and BUSCO version v4 (Simão et al., 2015), respectively. For detection of the small plasmids that may be missed by the initial assembly, the Unicycler hybrid assembly was used with the parameter mode set to “bold.” Candidate plasmids from the Unicycler were subsequently verified by mlplasmids (Arredondo-Alonso et al., 2018) and PlasFlow programs (Krawczyk et al., 2018). The plasmids verified by at least two programs were retrieved.

### WGS characterization, gene functional annotation, and comparative genome analysis

Upon the completion of the *de novo* assembly process, the WGS was characterized through the NCBI prokaryotic genome analysis pipeline for predicting protein-coding genes and genes for noncoding RNAs, e.g., rRNA and tRNA (Tatusova et al., 2016). In addition, the WGS was then characterized using different bioinformatic tools. For AMR gene prediction, the WGS in FASTA format was uploaded and analyzed under default settings (>90% identity and 60% minimum length) in the web server ResFinder 4.1^1^ (accessed on: 6 August 2022) (Florensa et al., 2022). For the detection of prophages, the WGS in FASTA format was uploaded and analyzed under default settings in the online web server PHASTER^2^ (accessed on: 29 March 2022; Arndt et al., 2016). Moreover, the sequence homology analysis was employed to identify genes potentially associated with AMR by searching all protein-coding genes against a dataset of 2,979 sequences of AMR genes from the comprehensive antibiotic resistance database [CARD;^3^ (accessed on: 7 September 2021)] (Alcock et al., 2020) using BLASTP (Altschul et al., 1990) with ≥ 80% identity and ≥ 80% coverage cut-off (EFSA, 2021). For gene functional annotation, the protein-coding genes were subsequently analyzed through eggNOG-mapper for clusters of orthologous gene (COG) annotation (Huerta-Cepas et al., 2017, 2019), PANNZER2 for gene ontology (GO) annotation (Törönen et al., 2018), and GhostKOALA for KEGG orthology (KO) annotation (Kanehisa et al., 2016). Additionally, a similarity search was performed using the GenBank database (non-redundant GenBank CDS translations + PDB + SwissProt + PIR + PRF excluding environmental samples from WGS projects, updated date: 31 May 2022, number of sequences: 483,768,206) and BLASTP available at^4^ (accessed on: 1 June 2022). Graphical genome visualization was generated by CGView (Grant and Stothard, 2008). For comparative genome analysis, all seven publicly available complete genomes of *L. pentosus* were obtained from the GenBank database (accessed on: 4 August 2022). The seven genomes included *L. pentosus* SLC13 (GCA_002211885.1), BGM48 (GCA_002850015.1), ZFM222 (GCA_003627295.1), ZFM94 (GCA_003627375.1), DSM 20314 (GCA_003641185.1), KW1 (GCA_023972895.1), and KW2 (GCA_023980805.1). The orthologous gene set was constructed to identify core-and pan-genome using CMG-biotools package version 2.2 (Vesth et al., 2013). Briefly, all predicted protein sequences were compared to each other using BLAST (Binnewies et al., 2005). In which, the two proteins are grouped as the same “gene family” when their BLAST alignment is at least 50% identical and covers 50% of the length of the longest sequence. The core-genome consists of gene families found in all analyzed genomes. The pan-genome is the entire set of gene families from all genomes in the comparison.

### Species identification and phylogenetic tree analysis

The 16S rRNA gene was used for initial species identification. Similarities of the 16S rRNA gene to the type/reference strain database were searched using the nucleotide basic local alignment search tool available at NCBI^4^ (accessed on: 1 June 2022). Phylogenetic tree analysis was performed using the Neighbor-joining method with the bootstrap test of 1000 replicates by MEGA11 (Saitou and Nei, 1987; Stecher et al., 2020; Tamura et al., 2021). The evolutionary distances in the units of the number of base substitutions per site were computed using the Kimura 2-parameter method (Kimura, 1980). The resulting phylogenetic tree was drawn by using Interactive Tree Of Life (iTOL) version 5 (Letunic and Bork, 2021). The species of 9D3 was then confirmed by calculating the average nucleotide identity (ANI) to the type strains of related species using JSpeciesWS (Richter et al., 2016). The strains used for ANI calculation included: *L. pentosus* DSM 20314^T^, *Lactiplantibacillus argentoratensis* DSM 16365^T^, *Lactiplantibacillus plantarum* subsp. *plantarum* ATCC 14917^T^, *Lactiplantibacillus xiangfangensis* LMG 26013^T^, and *Lacticaseibacillus fabifermentans* DSM 21115^T^. The ANI value of ≥ 95% was used as a criterion to confirm the species identification (Richter and Rosselló-Móra, 2009). In addition, the WGS similarities expressed as digital DNA–DNA hybridization (dDDH) scores were calculated using the Type (Strain) Genome Server (Meier-Kolthoff and Göker, 2019). For dDDH, a 70% threshold was used as the criterion for species designation (Wayne et al., 1987).

### *In vitro* tests for hemolytic activity, bile-salt deconjugation activity, and antimicrobial susceptibility

The hemolytic activity was identified by observing the zone around the growth on Columbia blood agar containing 5% sheep blood (Oxoid Ltd., United Kingdom) after being anaerobically incubated at 37°C for 48 h. The bile salt deconjugation activity was determined by observing the precipitation in or around the growth on MRS containing 0.5% taurodeoxycholic acid, or 0.5% glycodeoxycholic acid after 72 h of incubation at 37°C under anaerobic conditions (Dashkevicz and Feighner, 1989). The resistance phenotype of *L. pentosus* 9D3 was investigated as recommended by the European Food Safety Authority (EFSA FEEDAP, 2018). The strain’s susceptibility to seven antimicrobials, i.e., ampicillin, gentamicin, kanamycin, erythromycin, clindamycin, tetracycline, and chloramphenicol was determined. The minimum inhibitory concentration (MIC) for each antimicrobial was evaluated using the microdilution method following the international standard (International Organization for Standardization (ISO) and International Dairy Federation (IDF), 2010). The MICs were determined in LSM broth [90% ISO-sensitest broth (Oxoid Ltd., United Kingdom): 10% MRS], after incubation of the strain at 37°C under anaerobic conditions (10% H_2_, 10% CO_2_, 80% N_2_) for 48 h.

### *In vitro* digestion

*In vitro* digestion consisted of oral, gastric, and small intestinal phases and was performed following the method previously described (Garrett et al., 1999; Thakkar et al., 2007) with some modifications. For the oral phase, the bacterium was incubated in basal salt solution (120 mM NaCl, 5 mM KCl, 6 mM CaCl_2_) containing 300 units/ml α-amylase, incubated at 37°C with agitation at 95 rpm for 5 min. For the subsequent gastric phase, the cell suspension was adjusted to pH 2.0 with HCl, and pepsin was added to the final concentration of 2 mg/ml. The suspension was incubated with agitation at 37°C for 1 h. For the subsequent small intestinal phase, the cell suspension was adjusted to pH 6.0 and porcine bile extract, pancreatin, and lipase were added to the final concentrations of 2.4, 0.4, and 0.2 mg/ml, respectively. The solution was incubated at 37°C with agitation at 95 rpm for 2 h. At the end of each digestion phase, the viable bacterial cell counts were determined by plating appropriate 10-fold dilutions onto MRS agar and incubating at 30°C for 48 h. The assay was performed in triplicate. A strain of commercial probiotic, *Lacticaseibacillus rhamnosus* GG (ATCC 53103) was used for comparison. Student’s *t*-test was performed to determine statistical differences between 9D3 and *L. rhamnosus* GG. A *p*-value of 0.05 was considered a significant difference.

### *In vitro* adhesion

The human colonic carcinoma cell line (Caco-2) was prepared according to the method previously described (Garrett et al., 1999; Chitchumroonchokchai et al., 2004) with some modifications. The Caco-2 cells were cultured in Dulbecco’s modified Eagle’s medium (DMEM) containing 15% heat-inactivated fetal bovine serum (FBS), 2.5 mM L-glutamine, 100 U/ml penicillin, 100 μg/ml streptomycin, and 25 μg/ml amphotericin B. The cells were maintained under a humidified atmosphere containing 5% CO_2_ at 37°C. The spent medium was changed with a fresh medium every other day until reaching 80% confluency. For the adhesion assay, 4 × 10^4^ cells were seeded into 6-well culture plates and maintained as previously described. After confluency, the cells were cultured in the complete medium containing 7.5% FBS for 11–14 days. The culture medium was replaced with antibiotic-free DMEM supplemented with 7.5% FBS 1 day before the experiment. For the adhesion reaction, the Caco-2 cells were washed and maintained in the DMEM basal medium (without FBS and antibiotics). After the *in vitro* digestion, the bacterial cell suspension was prepared in the same basal medium and added to the Caco-2 cells, then incubated at 37°C in an incubator containing 5% CO_2_ for 1 h. The Caco-2 cells were then washed twice with sterile PBS to remove non-adhering bacteria, and treated with 0.05% TritonX-100 in sterile PBS to release the attached cells. The number of viable bacteria adhering to the Caco-2 cells was determined by plating appropriate 10-fold dilutions onto MRS agar. Percent adhesion was calculated as the ratio of the number of bacterial cells that remained attached to the total number of bacterial cells added initially to each well in the unit of CFU/well (Argyri et al., 2013). The assay was performed in triplicate. *L. rhamnosus* GG was used for comparison. Student’s *t*-test was performed to determine statistical differences between 9D3 and *L. rhamnosus* GG. A *p*-value of 0.05 was considered a significant difference.

## Results and discussion

### WGS of *Lactiplantibacillus pentosus* 9D3

**Figure 1:**
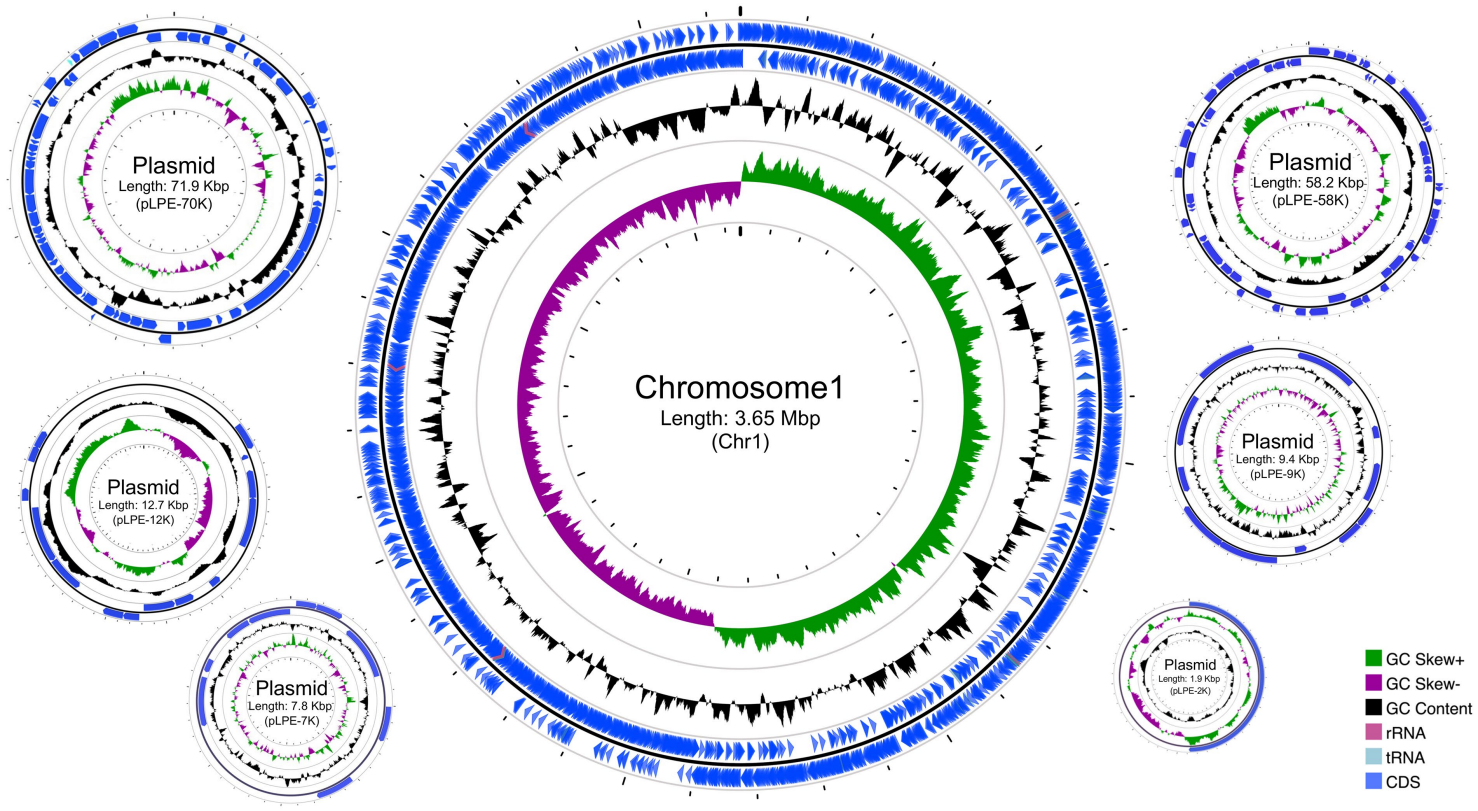
Circular genome maps of *Lactiplantibacillus pentosus* 9D3. From outside to center: Rings 1 and 2 demonstrate the protein-coding genes (CDS) (blue), tRNA (light blue), and rRNA (light purple) on the forward and reverse strands, respectively; ring 3 represents the GC content plot; ring 4 represents the positive and negative GC skew in green and purple, respectively.

A highly accurate and complete WGS of *L. pentosus* 9D3 was obtained. The complete genome has a total size of 3.81 Mbp and a GC content of 46.09%. This genome consists of a 3.65-Mbp chromosome and six plasmids in sizes ranging from 1.9 to 71.9 Kbp (Figure 1). A total of 3,456 genes were predicted with an average length of 923.9 bp, and these occupied 83.8% of the genome. Among them, 3,356 genes were predicted as protein-coding genes and the remaining 100 genes were tRNA (80 genes), rRNA (16 genes), and other noncoding RNA (4 genes).

The genome size and gene number of the 9D3 strain were similar to those of *L. pentosus* strains isolated from fermented olives, such as IG4 (3.81 Mbp, 3,522 genes) and MP-10 (3.70 Mbp, 3,558 genes) with very similar GC contents, varied from 45.97 to 46.32% (Abriouel et al., 2017a; Calero-Delgado et al., 2018). Nevertheless, the 9D3 strain showed a larger genome size and higher gene number than *L. pentosus* KCA1 isolated from a vaginal source (3.4 Mbp, 2,992 genes) (Anukam et al., 2013). This may be due to the presence of plasmids in *L. pentosus* 9D3 (6 plasmids) similar to IG4 (6 plasmids) and MP-10 (5 plasmids), while the KCA1 has no plasmid. The variations in the genome size and gene number may reflect different environmental nitches of the strains.

In addition, four potential prophages, including three intact prophages ranging from 40.8 to 47.7 Kbp integrated into the chromosome, and one incomplete prophage located in the plasmid pPLE-58K were found in the 9D3 genome (Table 1). The presence of intact prophages in a range of 1–5 per genome is found to be common in lactobacilli strains (Pei et al., 2021). For *L. pentosus*, previous studies reported the presence of two and four intact prophages in strains isolated from fermented olives and fermented sausages, respectively (Abriouel et al., 2017a; Stergiou et al., 2021). This is in line with our result in which three intact prophages were found in the genome. The presence of prophages in the bacterial genome may confer fitness to the harsh environment (Pei et al., 2021).

**Table 1:** Potential prophages detected in *L. pentosus* 9D3 genome.

Region	Location	Region length	Completeness	Region position
1	Chromosome	40.8 Kbp	Intact	633,578–674,451
2	Chromosome	45.1 Kbp	Intact	716,363–761,503
3	Chromosome	47.7 Kbp	Intact	2,440,532–2,488,313
4	Plasmid pPLE-58K	8.2 Kbp	Incomplete	30,938–39,208

### Species identification and phylogenomic profiling of *Lactiplantibacillus pentosus* 9D3

**Figure 2:**
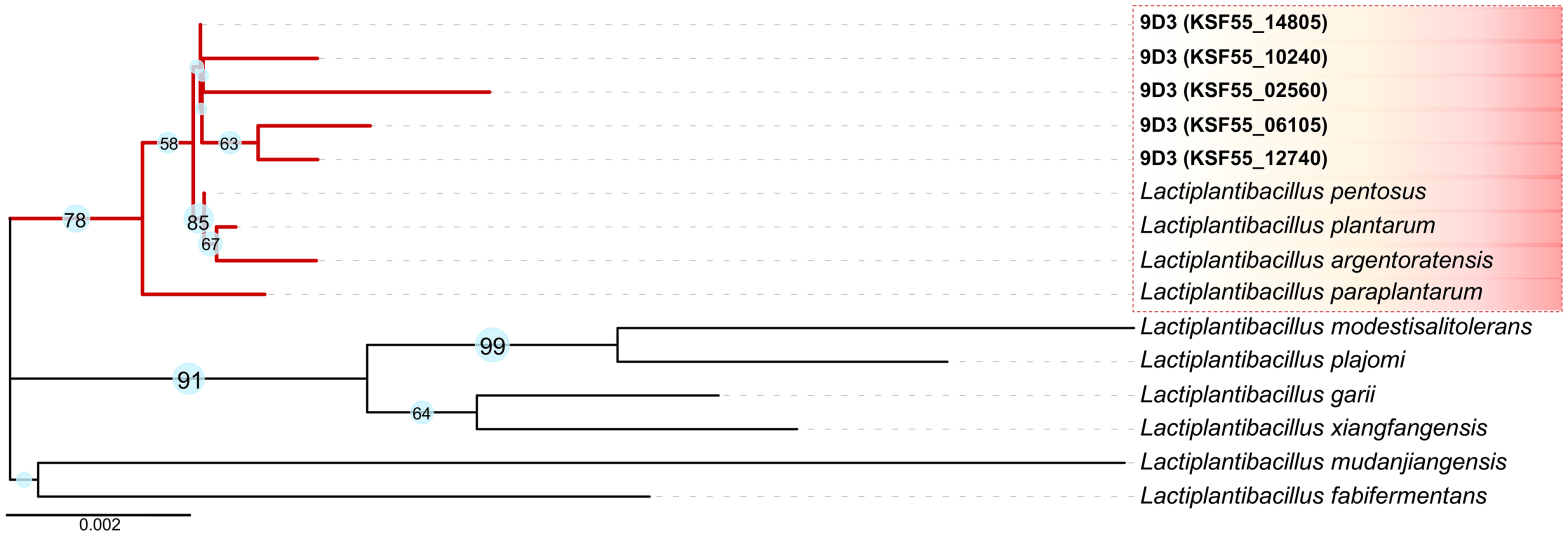
16S rRNA gene marker analysis of the 9D3 strain and the selected type strains of closely related species. The phylogenetic tree was constructed by the Neighbor-Joining method with 1,000 bootstrap replicates using MEGA11 (Saitou and Nei, 1987; Stecher et al., 2020; Tamura et al., 2021). Evolutionary distances in the units of the number of base substitutions per site were computed using the Kimura 2-parameter method (Kimura, 1980), and the visualization of the phylogenetic tree was drawn by iTOL version 5 (Letunic and Bork, 2021), in which only bootstrap values ≥ 50% are shown next to the branches.

For initial species identification, five copies of the 16S rRNA gene were identified in the 9D3 genome. These nucleotide sequences were highly similar but not identical, with 98.85–99.81% identity to each other (Supplementary Table S1). The sequences showed the highest sequence similarity to those of *L. pentosus* and *L. plantarum* (99.61–99.80%), and *L. paraplantarum* (99.47–99.67%); (Supplementary Table S2). A phylogenetic tree created from the 16S rRNA gene sequences of the 9D3 strain and the type strains of closely related species is shown in Figure 2. From the high sequence similarities of the 16S rRNA gene among several closely-related species in this group, the species identification of the 9D3 strain is inconclusive. For definitive species identification, the 9D3 strain showed the highest ANI value and dDDH score for the type strain of *L. pentosus* at 97.24 and 80.6%, respectively. Both values were above the species cut-off values of 95% for ANI and 70% for dDDH. In contrast, the other species indistinguishable by the 16S rRNA gene marker analysis showed clear distances to the strain with much lower ANI and dDDH scores, as detailed in Supplementary Table S3. Therefore, the 9D3 strain was unambiguously identified as *L. pentosus*.

### Gene function annotation and comparative analysis of *Lactiplantibacillus pentosus* 9D3 genome

**Figure 3:**
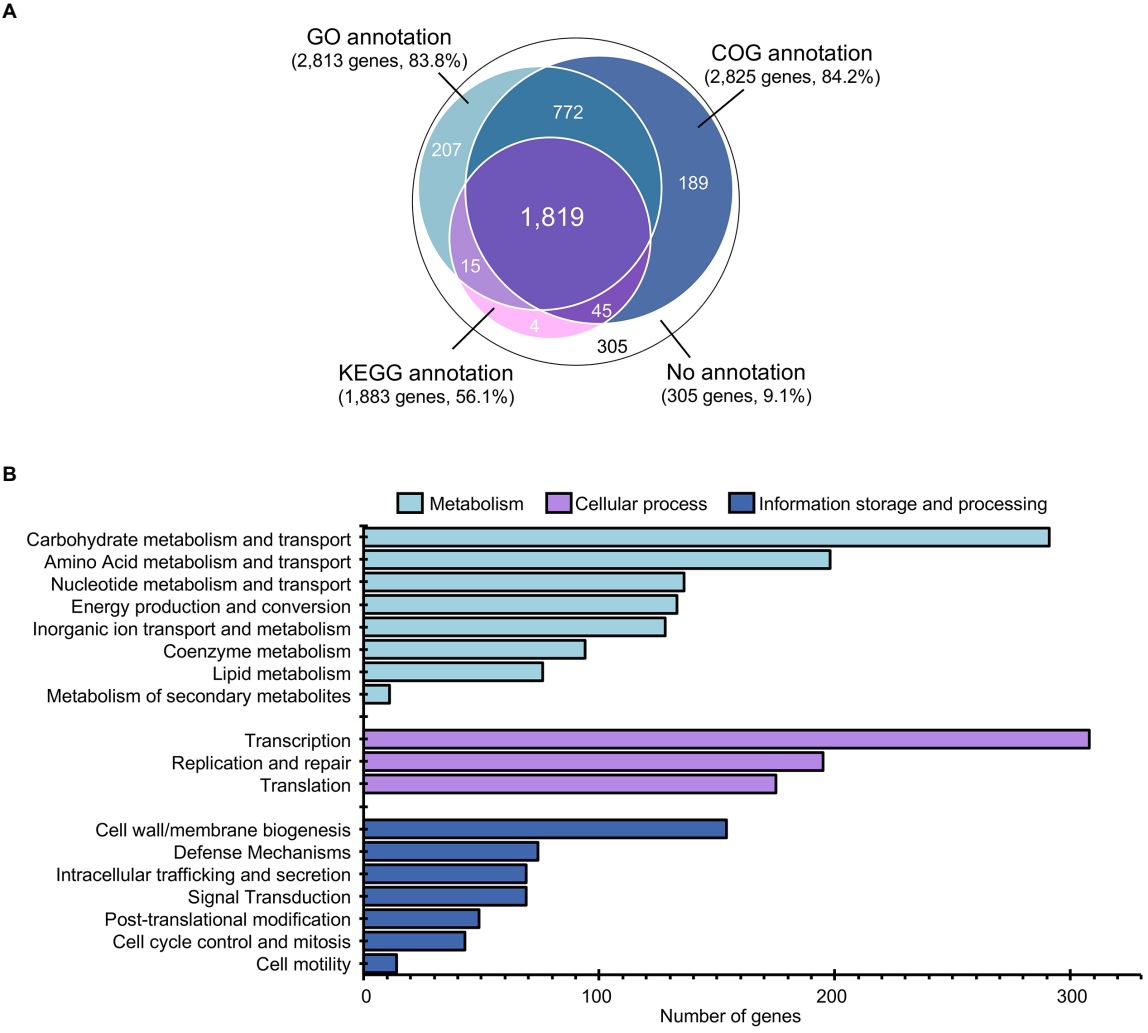
Annotation and gene function analysis of *L. pentosus* 9D3 genome. **(A)** Numbers of annotated genes from different databases. **(B)** Distribution of annotated genes across the different COG functional categories.

Among the 3,356 protein-coding genes, 3,031 genes could be assigned with functions regarding the different protein databases, i.e., COG (2,825), GO (2,813), and KEGG (1,883), as shown in Figure 3A and detailed in Supplementary Table S4. Analysis of gene function distribution across the different COG functional categories revealed that metabolism-related genes were the most abundant, accounting for 37.8% of the total COG annotation. Among them, the majority were involved in the metabolism and transport of carbohydrates, as shown in Figure 3B. This finding is in agreement with a previous study, which showed that *L. pentosus* is a versatile and flexible species that can grow on a wide variety of carbon sources (Abriouel et al., 2017b).

**Figure 4:**
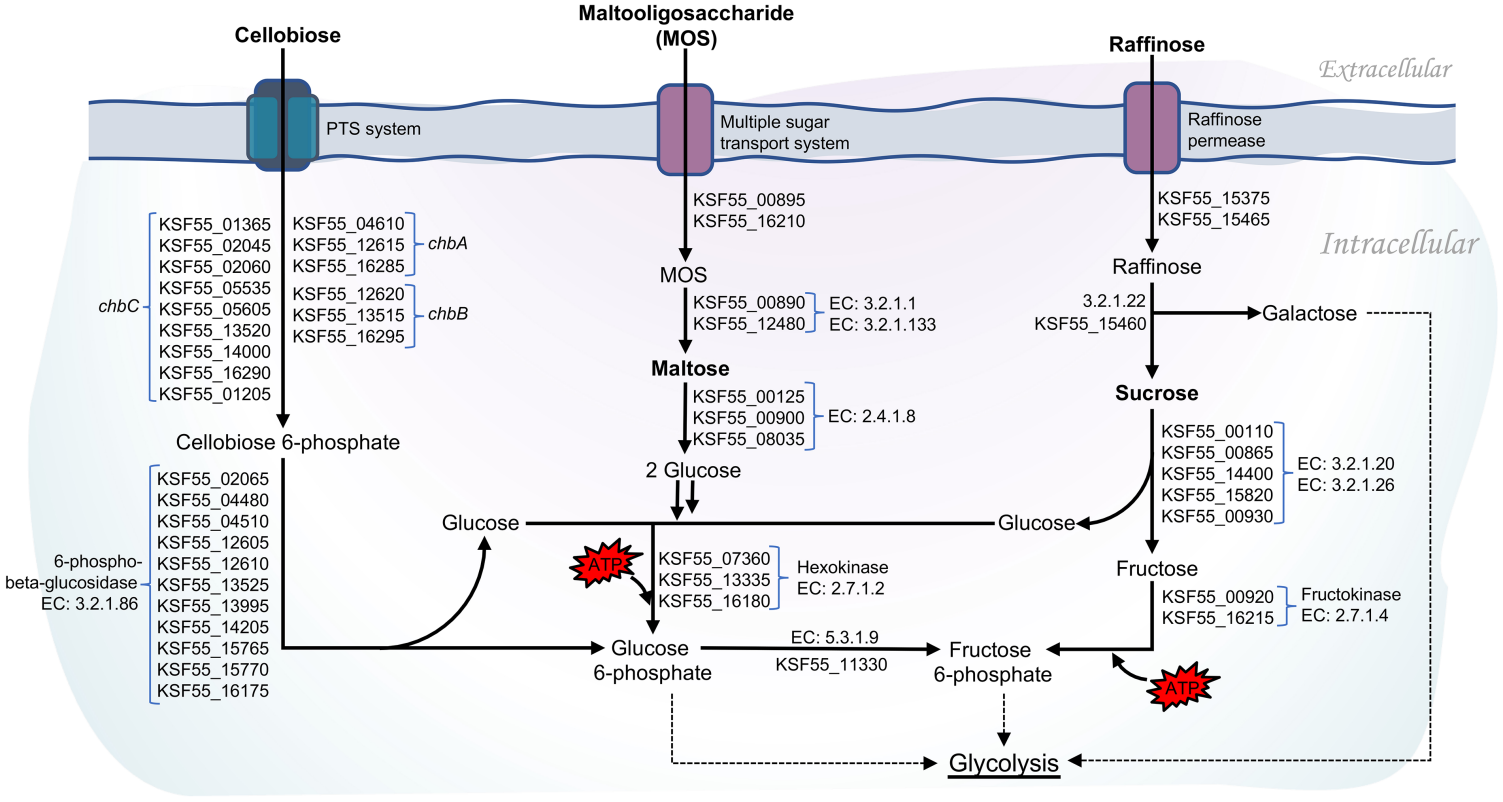
Pathways of carbohydrate utilization in *L. pentosus* 9D3.

Intriguingly, as many as 58 genes encoded for phosphotransferase systems (PTS) were identified in the 9D3 strain (Supplementary Table S5). The PTS catalyzes the phosphorylation of the incoming carbohydrates concomitant with their translocation across the cell membrane (McCoy et al., 2015). During the translocation process by PTS, the glycolytic conversion of phosphoenolpyruvate to pyruvate is activated, thereby increasing the production of glycolytic and TCA cycle intermediates (Gosset, 2005). The presence of PTS in *L. pentosus* may increase carbon transport capacity and thus enhance bacterial growth (Abriouel et al., 2017b). Notably, eleven copies of 6-phospho-beta-glucosidase (EC: 3.2.1.86), which hydrolyzes cellobiose 6-phosphate to yield glucose 6-phosphate and glucose, were identified in *L. pentosus* 9D3 genome (Figure 4) in addition to the large number of genes associated with PTS for cellobiose (15 genes). Compared to other disaccharides such as maltose and sucrose, the 6-phospho-beta-glucosidase pathway required only one ATP to metabolize cellobiose for glycolysis (Figure 4). This pathway is more energy efficient for disaccharide metabolism. Thus, the redundant cellobiose PTS and 6-phospho-beta-glucosidase genes suggest that *L. pentosus* 9D3 is well-adapted for growth in a cellobiose-rich environment. Besides, the prevalence of genes coding for disaccharides, monosaccharides, and polyols transport proteins supports that 9D3 is capable of utilizing a wide range of carbohydrates. These included sucrose, lactose, maltose, mannose, fructose, arabinose, galactitol, sorbitol, mannitol, and inositol (Supplementary Table S5). In addition, the genes involved in the transport and utilization of prebiotics such as maltooligosaccharide and raffinose were also identified, as shown in Figure 4. These results supported the versatility of the strain to survive and adapt to various environments, including the gastrointestinal (GI) environment, where simple sugars are scarce and non-digestible sugars are the main nutrient source.

**Figure 5:**
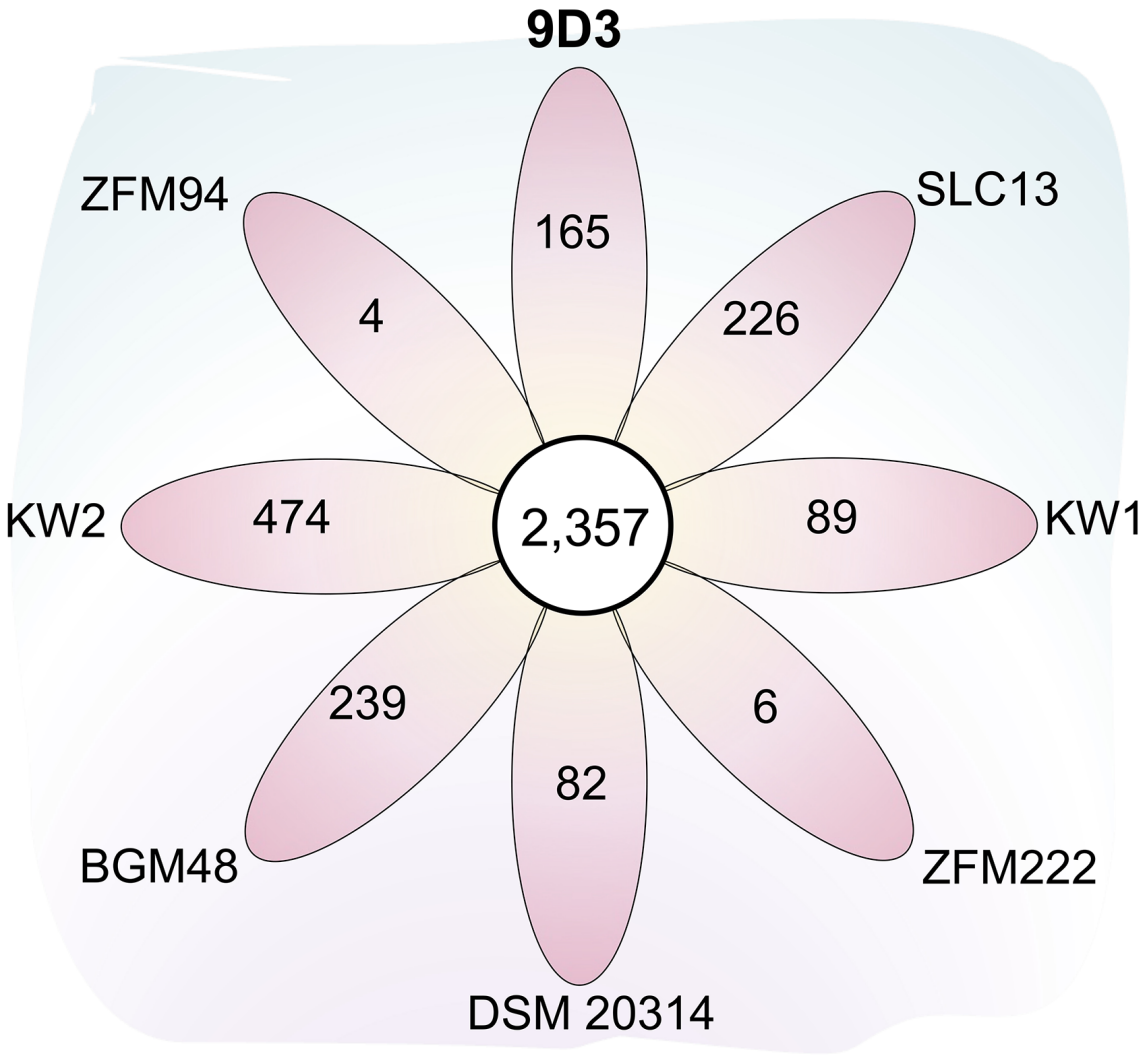
Flower plot diagram showing the numbers of core and unique genes among eight *L. pentosus* strains. The central circle shows the number of core gene families common to all strains while the petals show the numbers of genes unique to each strain. Abbreviated strain names are given outside each petal.

In comparison with the other seven *L. pentosus* strains, 2,357 and 4,837 gene families were identified as core-genome and pan-genome, respectively (Figure 5). A total of 165 genes were unique for *L. pentosus* 9D3 (Supplementary Table S6). Interestingly, a gene responsible for GABA production, i.e., glutamate decarboxylase (KSF55_17165: *gadA*) and secretion, i.e., glutamate-GABA antiporter (KSF55_17160: *gadC*) were uniquely identified on the pLPE-70 K plasmid of 9D3. This glutamate decarboxylase (GAD) system converts glutamate into GABA and helps to maintain cytosolic pH homeostasis. The system was shown to play a significant role in conferring acid resistance and allows LAB to survive in acidic environments (Wu et al., 2017). Moreover, the localization of the GAD genes in the plasmid infers a previous event of horizontal gene transfer of these genes into the genome of *L. pentosus* 9D3, providing the unique GABA-producing property of the strain when compared to the other analyzed *L. pentosus*. The genes related to the GABA-producing property of 9D3 exhibited high sequence similarity and synteny to *Lactiplantibacillus plantarum* KB1253, a GABA-producing reference strain (Nakatani et al., 2019), Figure 6. The result suggested that the GABA biosynthesis genes in 9D3 may be acquired from, or have a common ancestor with those of the GABA-producing *L. plantarum*. Since the GABA-producing capability of 9D3 is associated with a plasmid, maintenance of the plasmid is crucial for the strain application concerning this property. On the other hand, this finding also opens the possibility to develop new GABA-producing cultures by allocating the GABA-producing plasmid of *L. pentosus* 9D3 to other desirable strains suitable for the specific application.

**Figure 6:**
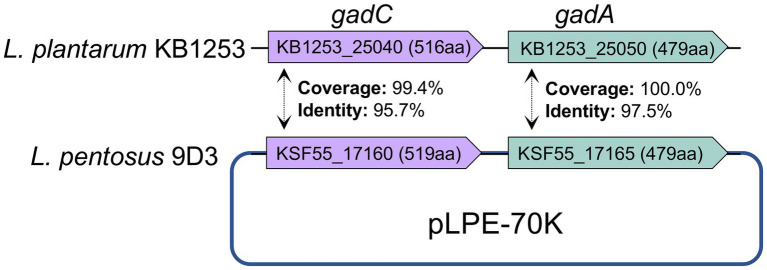
Comparison of genes related to GABA production of *L. pentosus* 9D3 and *L. plantarum* KB1253, a GABA-producing reference strain. *gadA*, glutamate decarboxylase; *gadC*, glutamate-GABA antiporter.

### Identification of genes implicated in the safety of *Lactiplantibacillus pentosus* 9D3

The safety of *L. pentosus* 9D3 was evaluated in terms of virulence and undesirable properties, AMR, and antimicrobial drug biosynthesis capability.

#### Virulence and undesirable properties

##### Hemolysis

Functional analysis based on KEGG annotation identified one bacterial toxin gene, *hlyIII*, encoding hemolysin III (KSF55_14680). The gene was found in many lactobacilli probiotic strains and concluded to involve no safety concerns. Hemolysis identified using sheep blood agar showed no visible clear zone around the growth, indicating the strain was nonhemolytic. However, a slight color change similar to the reaction exhibited by a probiotic control strain, *L. plantarum* 299 V was observed (Supplementary Figure S1). The slight color change from red to yellow around the growth was considered a result of acidity produced by the strain rather than the activity of toxins. Therefore, it was concluded that the *L. pentosus* 9D3 strain is safe in this aspect.

##### D-lactate formation

The result of the KEGG pathway analysis showed that the 9D3 genome contained a copy of the gene encoding for lactate racemase (EC: 5.1.2.1; KSF55_00515) and two copies of D-lactate dehydrogenase (EC: 1.1.1.28; KSF55_04355 and KSF55_09370), indicating its capability to convert L-lactate to D-lactate. D-lactate production is a property universally expressed in LAB since it is an essential component in cell wall peptidoglycan and is considered an intrinsic property of LAB. Specific concern on this property was due to historical records of D-lactic acidosis associated with probiotic consumption. However, this condition was very rare and occurred only in specific sub-populations, e.g., patients with carbohydrate malabsorption conditions that failed to control their carbohydrate consumption (Chokesajjawatee et al., 2020). This property was concluded to be of no safety concern for the general population.

##### Bile salts deconjugation

Identification of the genes involved in bile salts deconjugation activity was performed by KEGG pathway analysis. No gene encoding for the enzyme choloylglycine hydrolase/bile salt hydrolase (BSH) (EC: 3.5.1.24) was found in the 9D3 genome. The absence of bile salts deconjugation activity was confirmed by the absence of the precipitates in or around the growth on MRS containing 0.5% taurodeoxycholic acid or 0.5% glycodeoxycholic acid (Supplementary Figure S2). It should be noted that lacking this ability should not be interpreted as bile intolerance since the two properties are unrelated (Moser and Savage, 2001). The undesirability of BSH activity was controversial. It was mentioned among other properties to be characterized for safety assurance but also listed among the main characteristics tested for probiotic properties (FAO/WHO, 2002). A comprehensive review by Begley et al. (2006) discussed the possible impact of the BSH activity on the host in both aspects. On the beneficial side, oral administration of BSH-positive probiotics has been shown to significantly reduce cholesterol levels in humans and mice. The deconjugated bile salts are less efficiently reabsorbed, hence increasing the demand for cholesterol for *de novo* synthesis of bile acids. Also, deconjugated bile salts are less efficient in the solubilization of lipids, including cholesterol, thereby reducing the absorption of fat and cholesterol in the gut. However, the reduced emulsification of dietary lipids may compromise normal lipid digestion and impair fatty acids and monoglycerides absorption. Another possible safety concern is related to secondary bile acids resulting from the subsequent modification of unconjugated bile salts. The secondary bile acids may cause DNA damage, promote colon cancer, impair colonic mucosal function, and promote gallstone formation (Begley et al., 2006).

##### Biogenic amines formation

Genes related to the formation of biogenic amines, i.e., lysine decarboxylase (EC: 4.1.1.18), ornithine/lysine decarboxylase (EC: 4.1.1.116), arginine decarboxylase (EC: 4.1.1.19), agmatinase (EC: 3.5.3.11), spermidine synthase (EC: 2.5.1.16), arginase (EC: 3.5.3.1), ornithine decarboxylase (EC: 4.1.1.17), histidine decarboxylase (EC: 4.1.1.22), tyrosine decarboxylase (EC: 4.1.1.25), and tryptophan decarboxylase (EC: 4.1.1.28) were not found in the 9D3 genome. Therefore, food produced using this strain is safe from biogenic amine accumulation.

#### AMR property

According to the *in vitro* antimicrobial susceptibility test, the 9D3 strain showed resistance to two antimicrobials, ampicillin and chloramphenicol, indicating acquired resistance to these two drugs, as shown in Table 2. The search for the genetic determinants responsible for the Amp^r^ Chl^r^ phenotype by sequence similarity search using the EFSA (2021) recommended a cut-off value of 80% identity, and 80% coverage yielded no predicted AMR genes from the CARD database (all hits are shown in Supplementary Table S7). Further similarity search using the ResFinder database with the default parameters (90% identity, 60% coverage) also returned no-hit for acquired AMR genes. However, the Hidden-Markov model profile-based KO annotation and KEGG mapping analysis identified 14 possible AMR genes, as listed in Table 3. The beta-lactamase and chloramphenicol acetyltransferase genes were identified as the genetic determinants conferring the resistance phenotype to ampicillin and chloramphenicol, respectively. To determine the transferability of these AMR genes, the locations of all 14 putative AMR genes were compared to those of the mobile elements, plasmids and prophages, in the 9D3 genome. We focused on these two mobile elements since they pose a more immediate risk for horizontal gene transfer through conjugation/transformation and transduction, whereas other mobile elements such as transposons translocate genes within the genome and require additional steps/vehicles before the genes can be transferred to other organisms.

**Table 2:** Antimicrobial resistance (AMR) phenotype of *L. pentosus* 9D3.

Antimicrobial	Test range (mg/L)	Cut-off (mg/L)^*^	MIC (mg/L)	Interpretation
Ampicillin	0.25–32	2	4	Resistance
Chloramphenicol	0.25–32	8	16	Resistance
Clindamycin	0.06–8	4	0.06	Sensitive
Erythromycin	0.03–4	1	1	Sensitive
Gentamicin	0.5–64	16	4	Sensitive
Kanamycin	2–256	64	64	Sensitive
Tetracycline	1–128	32	32	Sensitive

* Epidemiological cut-off values according to EFSA FEEDAP (2018).

**Table 3:** List of putative AMR genes identified by KEGG annotation.

Gene ID	Gene name	KO	Confer resistance to	Function
KSF55_00370	*tetM, tetO*	K18220	Tetracycline	GTP-binding protein
KSF55_01045	*msrA, vmlR*	K18231	Macrolide	ATP-binding cassette domain-containing protein
KSF55_01670	*mef*	K08217	Macrolide	MFS transporter
KSF55_11770	*mef*	K08217	Macrolide	MFS transporter
KSF55_12535	*mef*	K08217	Macrolide	MFS transporter
KSF55_08305^*^	*catA*	K19217	Phenicol	Chloramphenicol acetyltransferase
KSF55_02240^*^	*penP*	K17836	beta-Lactam	Serine hydrolase
KSF55_04885	*vanY*	K07260	Vancomycin	D-alanyl-D-alanine carboxypeptidase family protein
KSF55_03890	*vanX*	K08641	Vancomycin	D-alanyl-D-alanine carboxypeptidase family protein
KSF55_06595	*norG*	K18907	Multidrug	PLP-dependent aminotransferase family protein
KSF55_11035	*abcA, bmrA*	K18104	Multidrug	ABC transporter, MdlB
KSF55_13340	*abcA, bmrA*	K18104	Multidrug	ABC transporter, MdlB
KSF55_14215	*mepR*	K18909	Multidrug	MarR family transcriptional regulator
KSF55_00435	*mepA*	K18908	Multidrug	MATE family efflux transporter

* Genes corresponding to the resistance phenotype.

Considering the locations of the AMR genes, it could be concluded that the genes responsible for the Amp^r^ Chl^r^ phenotype of 9D3 have a low risk of transfer since neither gene is associated with plasmids or prophages. Except for one, the rest of the putative AMR genes were not associated with any of the mobile elements of concern. The remaining AMR gene, KSF55_11035 (located at 2,443,645 bp - 2,445,408 bp), was found within an intact prophage region, raising concern about the possibility of transfer. From a similarity search using the GenBank database, the translated amino acids from this gene had 100% identity to an ABC transporter ATP-binding protein/permease, multidrug resistance-like ATP-binding protein (MdlB) of *L. pentosus* (accession number: WP_152705378). Although the gene was identified as a putative drug transporter, it was not confirmed experimentally. In contrast, a previous study reported that an *E. coli* strain transformed with a plasmid carrying the *mdlB* gene exhibited no increase in the resistance phenotype (Nishino and Yamaguchi, 2001). Since the strain showed no resistance to other antimicrobials except for the Amp^r^ Chl^r^ phenotype and the search using other AMR databases did not identify this gene as AMR associated with pathogens, we concluded that this transport gene might have other functions not related to AMR. Therefore, we concluded that the chance of transferring these AMR determinants to other organisms is low even though the strain was resistant to ampicillin and chloramphenicol.

#### Antimicrobial drug biosynthesis

Microbial culture used in food and feed should not produce any antimicrobial drug of clinical importance. This requirement aims to avoid the creation of a selective environment for the emergence of new AMR strains (Pariza et al., 2015). The biosynthesis pathways for critically important antimicrobial drugs, as identified in the World Health Organization’s complete list of critically important antimicrobials (WHO CIA list; WHO, 2019), were assessed *in silico*. No complete pathways for any of the CIAs were found in the 9D3 genome. A detail of the pathways analyzed and search results are shown in Supplementary Table S8. Historically, the species *L. pentosus* has never been noted for its ability to produce antimicrobial drugs of medical importance. Therefore, we concluded that *L. pentosus* 9D3 poses no safety concern in this regard.

### Evaluating the probiotic potential of *Lactiplantibacillus pentosus* 9D3

To exert its health benefit and function as a probiotic, the bacterium should be able to survive and remain within the host’s target site, i.e., the GI tract in the case of probiotics intentionally add to the food system. The potential of *L. pentosus* 9D3 to be used as a probiotic for food was assessed by its ability to survive during transit through a GI tract and its ability to adhere to intestinal epithelial cell line Caco-2.

#### Survival of *Lactiplantibacillus pentosus* 9D3 in the GI tract conditions

This *in vitro* study tested the ability of *L. pentosus* 9D3 to survive conditions mimicking the environment the bacterium is expected to encounter, from the oral phase to the intestinal phase. *L. pentosus* 9D3 survived well at the oral digestion stage, which contains α-amylase enzymes, and the viable counts remained high at 9.37 ± 0.03 log CFU/ml compared with pre-digestion (9.38 ± 0.04 log CFU/ml; Table 4). The strain also exhibited good survivability in the hostile stomach condition, i.e., pH 2.0 in the presence of digestive enzyme pepsin for 1 h, with approximately 1 log reduction. In the intestinal phase, where the acid-stressed cells encountered several digestive enzymes and bile salts as well as a slightly acidic condition at pH 6.0, the 9D3 strain showed moderate survivability, with approximately 2.4 log reduction. This result was in concert with an earlier study in which a similar log reduction among *L. pentosus* strains, 0.5–2 log in simulated gastric juice, and 1–3 log in simulated intestinal fluid were reported (Guantario et al., 2018). This response was comparable to that of the popularly used probiotic strain *L. rhamnosus* GG.

**Table 4:** Survival of *L. pentosus* 9D3 after *in vitro* digestion.

Strain	Pre-digestion (log CFU/ml)	Oral phase (log CFU/ml)	Gastric phase (log CFU/ml)	Small intestinal phase (log CFU/ml)
*L. pentosus* 9D3	9.38 ± 0.04^a^	9.37 ± 0.03^a^	8.33 ± 0.03^a^	5.95 ± 0.12^a^
*L. rhamnosus* GG	9.68 ± 0.13^b^	9.61 ± 0.03^b^	8.37 ± 0.02^a^	5.94 ± 0.49^a^

The same letter in each column represents no significant difference (value of *p* > 0.05). All data were mean values of triplicate analyzes (*n* = 3) ± standard deviation.

#### Adhesion ability of *Lactiplantibacillus pentosus* 9D3

Another important property of a probiotic is the ability to adhere to the intestinal mucosa. This property is important because it prevents the rapid elimination of probiotic cells by peristalsis and thus represents an ecologically competitive advantage in the ecosystem of the GI tract. A simple *in vitro* intestinal tissue model Caco-2 was used in this study. The cells were grown in culture to form a homogeneous polar monolayer of mature enterocytes resembling the tissue of the small intestine. After going through *in vitro* digestion, bacterial cells were used for the adhesion experiment to resemble the actual conditions the bacterial cells may encounter *in vivo*. *L. pentosus* 9D3 showed good adhesion ability, in which *ca.* 50% of the bacterial cells were attached to the Caco-2. This adhesion ability is comparable to the positive control *L. rhamnosus* GG (Table 5). The attachment ability was shown to be strain-specific and can be varied widely. The adhesion ability of *L. pentosus* strains at comparable levels to that of *L. rhamnosus* GG was also reported in the study of Argyri et al. (2013).

**Table 5:** Adhesion of *L. pentosus* 9D3 to Caco-2 cells.

Strain	Total number of added bacteria (log CFU/well)	Total number of adhered bacteria (log CFU/well)	% Adhesion
*L. pentosus* 9D3	5.91 ± 0.27^a^	5.61 ± 0.31^a^	49.69 ± 4.57^a^
*L. rhamnosus* GG	5.68 ± 0.04^a^	5.41 ± 0.07^a^	53.18 ± 3.67^a^

The same letter in each column represents no significant difference (value of *p* > 0.05). All data were mean values of triplicate analyzes (*n* = 3) ± standard deviation. Percent adhesion was calculated as the ratio of the number of bacterial cells that remained attached to the total number of bacterial cells added initially to each well (CFU/well).

Overall, *L. pentosus* 9D3 exhibited the basic requirement properties of a probiotic, i.e., capable to survive the digestive condition and attachment to the intestinal epithelial. However, according to the Guidelines for the Evaluation of Probiotics in Food (FAO/WHO, 2002), the health benefit of the strain should be identified *in vivo*. Therefore, the benefit to the host after administration of this strain is the subject for further investigation.

## Conclusion

In the present study, *L. pentosus* 9D3, a GABA-producing strain isolated from Thai pickled weed was evaluated for its safety and probiotic properties. Through WGS analysis, *L. pentosus* 9D3 was shown to be safe and free from functional and transferable AMR genes. The genome contains no genes related to the production of toxins, biogenic amines, and antimicrobial drugs of clinical importance. In addition, the genetic determinants conferring the GABA-producing capability of the strain were identified. A precaution about the acquired nature of this property is noted. The probiotic potential of the strain was shown by its good survivability in the simulated human GI tract conditions and good adhesion ability. The results can be used to support further investigation to identify the strain and the food it produces as probiotic products.

## Data availability statement

The datasets presented in this study can be found in online repositories. The names of the repository/repositories and accession number(s) can be found at: https://www.ncbi.nlm.nih.gov/bioproject/739022, NCBI-SRA repository under the BioProject accession number: PRJNA739022.

## Author contributions

NC and NR conceptualization. NC, PS, WM, and NR methodology. CS and WV resources. NC, WM, and NR data curation. NC, PS, and NR visualization. NC and NR writing original draft preparation. NC, CS, and NR writing review and editing. CS project administration. WV and CS funding acquisition. NR and NC supervision. All authors have read and agreed to the published version of the manuscript.

## Funding

This study was funded by The Agricultural Research Development Agency (ARDA) of Thailand (grant number: 2556NRCT512244); Institute of Nutrition, Mahidol University; the National Center for Genetic Engineering and Biotechnology, National Science and Technology Development Agency, Thailand (Project Code P-17-52209).

## Conflict of interest

The authors declare that the research was conducted in the absence of any commercial or financial relationships that could be construed as a potential conflict of interest.

## Publisher’s note

All claims expressed in this article are solely those of the authors and do not necessarily represent those of their affiliated organizations, or those of the publisher, the editors and the reviewers. Any product that may be evaluated in this article, or claim that may be made by its manufacturer, is not guaranteed or endorsed by the publisher.

## Footnotes

^1^ https://cge.cbs.dtu.dk/services/ResFinder

^2^ https://phaster.ca

^3^ https://card.mcmaster.ca

^4^ https://blast.ncbi.nlm.nih.gov
