# Supplementary material for: Whole-genome sequence analysis for evaluating the safety and probiotic potential of Lactiplantibacillus pentosus 9D3, a gamma-aminobutyric acid (GABA)-producing strain isolated from Thai pickled weed
Source: Front Microbiol. 2022 Sep 23;13:969548. doi: 10.3389/fmicb.2022.969548 (PMC9539741; doi:10.3389/fmicb.2022.969548)
Supplement: Supplementary file 2 [file Presentation_1.PDF]

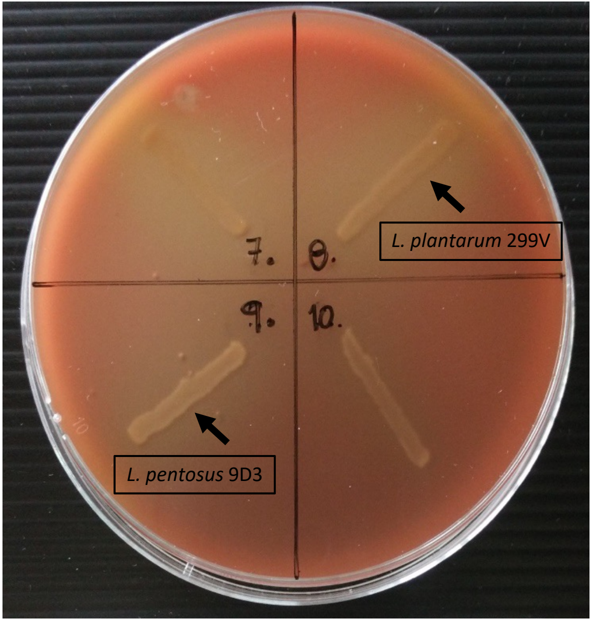

Supplementary Figure S1. Hemolysis activity of *L. pentosus* 9D3 on Columbia agar containing 5% sheep blood compared to a probiotic control strain *L. plantarum* 299V after incubated at 37°C for 48 h, anaerobic condition.

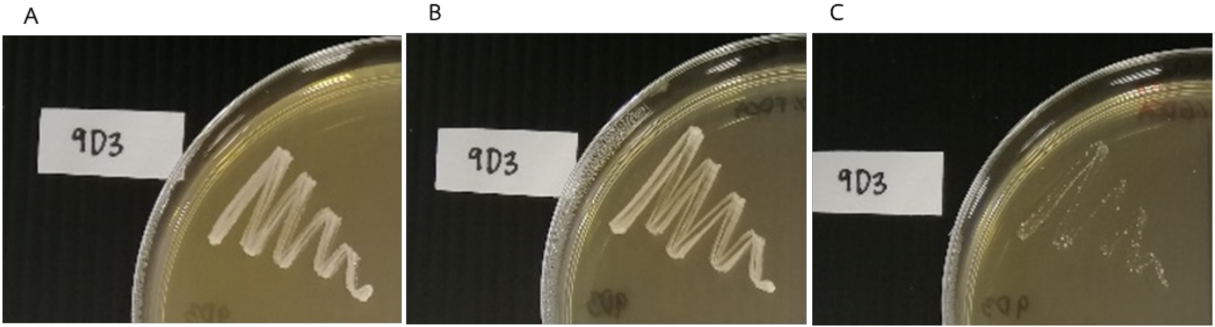

Supplementary Figure S2. Bile salts deconjugation activity of *L. pentosus* 9D3. Growth on MRS (A), MRS containing 0.5% taurodeoxycholic acid (B), and MRS containing 0.5% glycodeoxycholic acid (C).
